# β_3_‐Adrenoceptor stimulation of perivascular adipocytes leads to increased fat cell‐derived NO and vascular relaxation in small arteries

**DOI:** 10.1111/bph.14433

**Published:** 2018-08-10

**Authors:** Charlotte E Bussey, Sarah B Withers, Sophie N Saxton, Neil Bodagh, Robert G Aldous, Anthony M Heagerty

**Affiliations:** ^1^ Institute of Cardiovascular Sciences, University of Manchester Manchester UK; ^2^ Environment and Life Sciences University of Salford Salford UK

## Abstract

**Background and Purpose:**

In response to noradrenaline, healthy perivascular adipose tissue (PVAT) exerts an anticontractile effect on adjacent small arterial tissue. Organ bath solution transfer experiments have demonstrated the release of PVAT‐derived relaxing factors that mediate this function. The present studies were designed to investigate the mechanism responsible for the noradrenaline‐induced PVAT anticontractile effect.

**Experimental Approach:**

*In vitro* rat small arterial contractile function was assessed using wire myography in the presence and absence of PVAT and the effects of sympathomimetic stimulation on the PVAT environment explored using Western blotting and assays of organ bath buffer.

**Key Results:**

PVAT elicited an anticontractile effect in response to noradrenaline but not phenylephrine stimulation. In arteries surrounded by intact PVAT, the β_3_‐adrenoceptor agonist, CL‐316243, reduced the vasoconstrictor effect of phenylephrine but not noradrenaline. K_v_7 channel inhibition using XE 991 reversed the noradrenaline‐induced anticontractile effect in exogenously applied PVAT studies. Adrenergic stimulation of PVAT with noradrenaline and CL‐316243, but not phenylephrine, was associated with increased adipocyte‐derived NO production, and the contractile response to noradrenaline was augmented following incubation of exogenous PVAT with L‐NMMA. PVAT from eNOS^−/−^ mice had no anticontractile effect. Assays of adipocyte cAMP demonstrated an increase with noradrenaline stimulation implicating Gα_s_ signalling in this process.

**Conclusions and Implications:**

We have shown that adipocyte‐located β_3_‐adrenoceptor stimulation leads to activation of Gα_s_ signalling pathways with increased cAMP and the release of adipocyte‐derived NO. This process is dependent upon K_v_7 channel function. We conclude that adipocyte‐derived NO plays a central role in anticontractile activity when rodent PVAT is stimulated by noradrenaline.

**Abbreviations**
eNOS: endothelial NOS
KPSS: high potassium physiological salt solution
l‐NMMA: *N* ^G^‐monomethyl‐l‐arginine
nNOS: neuronal NOS
PDRF: PVAT‐derived relaxing factor
PSS: physiological salt solution
PVAT: perivascular adipose tissue
XE 991: 10,10‐bis(4‐pyridinylmethyl)‐9(10*H*)‐anthracenone dihydrochloride

## Introduction

Perivascular adipose tissue (PVAT) surrounds virtually all blood vessels and exerts an anticontractile effect in response to various vasoconstrictor agonists (Gao *et al*., 2007; Greenstein *et al*., 2009). The mechanisms by which PVAT exerts this effect are the subject of intense investigations, and what is clear already from organ bath studies is that adipocytes release transferable relaxing factor(s) (Lohn *et al*., 2002; Greenstein *et al*., 2009). However, the precise identity of these is unclear.

Recent evidence has indicated that http://www.guidetopharmacology.org/GRAC/ObjectDisplayForward?objectId=30 may be involved in the release of PVAT‐derived relaxing factors (PDRFs) provoked by sympathomimetic hormones: Weston *et al*. (2013) demonstrated that the specific β_3_‐agonist, http://www.guidetopharmacology.org/GRAC/LigandDisplayForward?ligandId=3462, induced endothelium‐independent but PVAT‐dependent myocyte hyperpolarization in rat mesenteric artery via the opening of potassium http://www.guidetopharmacology.org/GRAC/ObjectDisplayForward?objectId=380.

Vascular smooth muscle cell potassium channels are key regulators of membrane potential, with increased or decreased activity resulting in hyperpolarization or depolarization, respectively, meaning that they act as ‘off switches’ in excitable cells and occupy a central position in the regulation of vascular tone (Ghatta *et al*., 2006). Myocyte membrane potential is hyperpolarized in the presence of PVAT, and substantial evidence points towards a role of potassium channels in the anticontractile mechanism. Pharmacological investigations have led to the identification of several potassium channel candidates including http://www.guidetopharmacology.org/GRAC/FamilyDisplayForward?familyId=81 (Fésüs *et al*., 2007; Schleifenbaum *et al*., 2010) and BK_Ca_ (Gao *et al*., 2005; Lynch *et al*., 2013), which appear to undergo differential activation depending on the vascular bed and species investigated. Collectively, these studies indicate the emerging importance of PVAT, which appears to exert its anticontractile effect through a variety of complex pathways that may be activated in parallel in response to different agonists.

Therefore, the present studies were designed to investigate the mechanisms responsible for the http://www.guidetopharmacology.org/GRAC/LigandDisplayForward?ligandId=484‐induced PVAT anticontractile effect. We report that β_3_‐adrenoceptor stimulation of adipocytes leads to Gα_s_ signalling, a rise in http://www.guidetopharmacology.org/GRAC/LigandDisplayForward?ligandId=2352 and K_v_7 channel activation. The PDRF is http://www.guidetopharmacology.org/GRAC/LigandDisplayForward?ligandId=2509.

## Methods

### Group sizes

For all experiments, the group size is provided within the figure legends. For all experiments, the number of animals is reported. Assays for cAMP and NO were performed in duplicate and an average for each animal used for calculation of mean data.

### Randomization

All rats used in this study were healthy adult male Sprague Dawley rats bred at Charles River, UK. Rats were randomly assigned to a numbered cage upon delivery from Charles River, and the animals were used when they reached an appropriate size (250–300 g). The pharmacological protocol to be used in each myograph bath was determined prior to mounting the vessels.

### Blinding

Experimental blinding was not used for this study as there was one core experimenter responsible for dissection, experimental protocols and analysis. In order to limit experimental bias, data analysis was not performed until experimental data sets were complete. Duplicate data from a minimum of five separate animals were required for all experimental protocols described.

### Normalization

Contractile responses are expressed as mean percentages of KPSS constriction ± SEM consistent with previously published data (Greenstein *et al*., 2009; Withers *et al*., 2011). This allowed for comparison in contractile changes independent of vessel size.

### Validity of animal model

Male Sprague Dawley rats are a well‐established model for the investigation of PVAT function and vascular contractility (Greenstein *et al*., 2009; Aghamohammadzadeh *et al*., 2013). The http://www.guidetopharmacology.org/GRAC/ObjectDisplayForward?objectId=1249‐deficient (eNOS^−/−^) mouse model is well published and commercially available. This model was chosen to confirm the role of eNOS in our proposed mechanism. We have previously shown comparable response of mouse and rat mesenteric arteries to adrenergic stimulation (Withers *et al*., 2011). Myography protocols detailed below have been validated within our laboratory for rat, mouse and human tissues (Greenstein *et al*., 2009). Antibodies which were selective for rat antigens were chosen for immunohistochemistry and Western blot studies.

### Ethical statement

Experiments involving the use of animals were performed in accord with the UK Animals (Scientific Procedures) Act 1986 under the authority of a valid project licence. Animal studies are reported in compliance with the ARRIVE guidelines (Kilkenny *et al*., 2010; McGrath and Lilley, 2015). The experiments were approved by The University of Manchester Ethical Review Process. All work was carried out in accordance with the Declaration of Helsinki.

### Animals

Healthy male Sprague Dawley rats (250–300 g; Charles River, Harlow, Hertfordshire, UK) were killed by CO_2_ inhalation with death confirmed by permanent cessation of the circulation by severing the diaphragm. The mesenteric bed was immediately removed and placed in ice‐cold physiological salt solution (PSS in mmol·L^−1^: 119 NaCl, 4.7 KCl, 1.17 MgSO_4_, 25 NaHCO_3_, 1.17 KH_2_PO_4_, 0.03 K_2_ EDTA, 5.5 glucose and 1.6 CaCl_2_).

Male eNOS‐deficient (eNOS^−/−^) mice were kindly provided by Dr Elizabeth Cottrell (Manchester, UK). eNOS^−/−^ mice were bred in‐house at the University of Manchester and used at 12‐week‐old. As a control for eNOS^−/−^ mice, age‐matched male C57BL6/j mice were purchased from Envigo, Belton, Leicestershire, UK. Mice were killed as described above. Mesenteric artery samples were obtained and studied as described below.

### Wire myography

Small mesenteric arteries (internal diameter: 200–300 μm) both with and without PVAT were dissected and mounted on two 40 μm wires in a wire myograph (Danish MyoTech, Aarhus, Denmark, Denmark). The vessels were gassed (95% air/5% CO_2_) and maintained in PSS at 37°C for 30 min before wall tension and diameter were normalized using a standardized procedure (Mulvany and Halpern, 1977). Isometric tension was continuously recorded (Chart 5, v5.5, AD Instruments, Oxford, Oxfordshire, UK). Vessels were challenged with 60 mmol·L^−1^ high potassium PSS (KPSS in mmol·L^−1^: 63.7 NaCl, 60 KCl, 1.17 MgSO_4_, 25 NaHCO_3_, 1.17 KH_2_PO_4_, 0.03 K_2_ EDTA, 5.5 glucose and 1.6 CaCl_2_) to establish viability, and functional endothelial integrity was assessed by relaxation to 10 μmol·L^−1^ http://www.guidetopharmacology.org/GRAC/LigandDisplayForward?ligandId=298 following stable constriction with 10·μmol·L^−1^ noradrenaline.

When investigating the role of the endothelium, endothelium was removed by inserting equine hair into the lumen. Success in removing endothelium was tested using 10 μmol·L^−1^ carbachol as above.

#### Pharmacological assessment

Pharmacological protocols were used to study the mechanisms involved in mediating the PVAT anticontractile effect. In each case, segments from the same artery were prepared with and without PVAT and incubated either with PSS as a vehicle control or PSS containing the pharmacological tool for 30 min. The role of β_3_‐adrenoceptor‐mediated signalling was evaluated by incubation of vessels with the β_3_‐adrenoceptor agonist, CL‐316243, or the β_3_‐adrenoceptor antagonist, http://www.guidetopharmacology.org/GRAC/LigandDisplayForward?ligandId=547. The contribution of NO to PVAT function was assessed by incubation with the non‐specific NOS inhibitor, *N* ^G^‐monomethyl‐l‐arginine (l‐NMMA) (Aghamohammadzadeh *et al*., 2013), and http://www.guidetopharmacology.org/GRAC/LigandDisplayForward?ligandId=8830, which has been shown to be selective for http://www.guidetopharmacology.org/GRAC/ObjectDisplayForward?objectId=1251 at a concentration of 100 nmol·L^−1^ (Babu and Griffith, 1998). Protocols investigated the contribution of K^+^ channels through incubation with the K_v_7 channel inhibitor – http://www.guidetopharmacology.org/GRAC/LigandDisplayForward?ligandId=2596 (Li *et al*., 2013).

Following incubation with pharmacological tools, concentration–responses were constructed through the cumulative addition of noradrenaline or phenylephrine to the myograph bath (1 × 10^−7^–3.51 × 10^−5^ mol·L^−1^). The use of these two vasoconstrictor agonists allowed the role of β‐adrenoceptor‐mediated signalling to be investigated, as noradrenaline has equal affinity for α and β‐adrenoceptors, whereas http://www.guidetopharmacology.org/GRAC/LigandDisplayForward?ligandId=485 has higher affinity for http://www.guidetopharmacology.org/GRAC/FamilyDisplayForward?familyId=4 (Minneman *et al*., 2015).

#### Solution transfer experiments

Bioassay experiments were used to study the release of relaxing factors from PVAT (Lohn *et al*., 2002; Gao *et al*., 2007; Greenstein *et al*., 2009). PVAT with and without its adjacent artery was used as a donor, whereas arteries stripped of PVAT were used as recipients. Both the PVAT and recipient arteries were stimulated with 30 μmol·L^−1^ noradrenaline, and once a stable constriction had developed, solutions were removed from both baths and transferred from the bath containing the donor PVAT to the recipient artery without PVAT. The change in tension in the recipient artery was recorded. In order to investigate the contribution of external calcium to the anticontractile mechanism, donor PVAT with and without arteries was stimulated in PSS with (1.6 mmol·L^−1^) and without calcium. In experiments where PVAT was incubated in calcium‐free PSS, the calcium concentration of the solution was restored prior to addition to the recipient artery.

#### Exogenous PVAT experiments

Concentration–responses to noradrenaline were also constructed in vessels devoid of PVAT but with PVAT suspended on a wire within the myograph bath (exogenous PVAT) to enable examination of the release of relaxing factors from PVAT in response to different vasoconstrictor agonists. In some experiments, exogenous PVAT was incubated with various pharmacological tools for 30 min before the return of PVAT to the myograph bath to enable further investigation of PVAT mechanisms. Exogenous PVAT was incubated with http://www.guidetopharmacology.org/GRAC/LigandDisplayForward?ligandId=5190 to examine the effects of Gαs activation, db‐cAMP to assess the role of cAMP, l‐NMMA and vinyl‐l‐NIO to explore the role of PVAT‐derived NO and XE 991 to investigate K_v_7 activation within PVAT.

### Stimulation of PVAT

PVAT samples (~200 mg) were dissected from small mesenteric arteries and incubated in HEPES buffer (pH 7.4, in mmol·L^−1^: 127 NaCl, 5.9 KCl, 1.2 MgSO_4_, 10 HEPES, 2.4 CaCl_2_ and 11.8 glucose) in the absence (control) or presence of noradrenaline (10 μmol·L^−1^) or CL‐316243 (10 μmol·L^−1^) for 30 min at 37°C. The solution surrounding the PVAT (supernatant) was removed, and both tissue and supernatant were snap frozen in liquid nitrogen and stored separately at −80°C until use.

### Western blot analysis

In order to investigate the effects of noradrenaline stimulation on downstream signalling within PVAT, Western blot analysis was performed on unstimulated and stimulated PVAT lysate. PVAT samples were homogenized in RIPA buffer containing protease and phosphatase inhibitors using a FastPrep‐24 5G instrument with lysing matrix 5 tubes. Protein concentrations were determined using a Bradford assay. Samples (40 μg of protein) were separated using SDS‐PAGE on a 12% stain‐free SDS‐PAGE gel (Bio‐Rad Laboratories, Watford, Hertfordshire, UK). Following electrophoresis, samples were transferred to a low‐fluorescence PVDF membrane using a Trans‐Blot Turbo system, and transfer was verified using stain‐free imaging. Non‐specific binding sites were blocked with 5% BSA in TBS‐Tween (0.1% Tween), followed by overnight incubation with primary antibody at 4°C. Specific primary antibodies against http://www.guidetopharmacology.org/GRAC/FamilyDisplayForward?familyId=474 (0.27 μg·mL^−1^), phosphorylated‐AMPKα (0.26 μg·mL^−1^), β_3_‐adrenoceptor (5 μg·mL^−1^) and eNOS (1 μg·mL^−1^) were used. This was followed by three 10 min washes in TBS‐Tween at room temperature and exposure to secondary antibody (donkey anti‐rabbit HRP, 0.8 μg·mL^−1^) for 1 h at room temperature. Protein reactivity was detected using Clarity Western ECL substrate chemiluminescent detection reagent and captured digitally using the ChemiDoc MP imaging system. Stain‐free technology was used as a normalization tool as it allowed normalization to total protein levels through quantification of signal intensity within the whole sample (Ferguson *et al*., 2005; Gurtler *et al*., 2013). The chemiluminescent signal intensity of a given protein was normalized to the relative quantification of the corresponding intensity of the total protein, and changes in expression are expressed as the fold increase over unstimulated controls, assigning a value of 1 to the control. Data from each replicate were normalized and then averaged across the replicates, and data are plotted as mean ± SEM.

### Immunohistochemistry

PVAT samples were placed in 4% paraformaldehyde for 18 h and subsequently processed to paraffin wax blocks prior to serial sectioning at 5 μm. Samples were de‐waxed using xylene and rehydrated with ethanol prior to immunostaining for http://www.guidetopharmacology.org/GRAC/ObjectDisplayForward?objectId=563 expression (4 μg·mL^−1^). Heat‐induced antigen retrieval was performed followed by blocking of endogenous peroxidase using 3% H_2_O_2_ and block of non‐specific binding sites using goat serum. Sections were incubated with anti‐K_v_7.4 antibody overnight, followed by 1 h incubation with Biotin‐SP conjugated anti‐rabbit secondary antibody (1.5 μg·mL^−1^) and detection using Vectastain ABC complex followed by the addition of 3,3′‐diaminobenzidine (DAB) solution to allow visualization of antibody binding. Images were captured using a colour camera (Leica DFC450) mounted on a microscope (Leica DM5000).

### cAMP assay

Following stimulation, PVAT samples were homogenized using a FastPrep‐24 5G instrument with lysing matrix 5 tubes in ice‐cold 0.1 mol·L^−1^ hydrochloric acid at a 1:5 ratio (w^.^v^‐1^) to inactivate any PDEs within the sample. Samples were then centrifuged at 1000 *g* for 10 min to remove insoluble cellular debris and neutralized with 1 mol·L^−1^ sodium hydroxide. Samples were diluted 100‐fold in calibrator diluent before intracellular cAMP levels were determined using the competitive enzyme immunoassay cAMP parameter assay kit.

### NO levels within PVAT supernatant

NO within PVAT supernatant was converted to detectable nitrite by 3 h incubation with nitrate reductase (0.17 U·mL^−1^), glucose‐6‐phosphate dehydrogenase (4.17 U·mL^−1^), glucose‐6‐phosphate (500 μmol·L^−1^) and NADPH (1.67 μmol·L^−1^) as described previously (Verdon *et al*., 1995). A Griess reagent kit was then used to determine total nitrite concentration.

### Total adiponectin ELISA of PVAT supernatant

Total http://www.guidetopharmacology.org/GRAC/LigandDisplayForward?ligandId=3726 levels in stimulated and non‐stimulated PVAT supernatant samples were measured using a commercially available ELISA kit. Stimulated and unstimulated supernatant samples were diluted 1:750 in calibrator diluent before the assay was performed according to the manufacturer's instructions.

### Data and statistical analysis

The data and statistical analysis comply with British Journal of Pharmacology guidelines (Curtis *et al*., 2018). GraphPad Prism (v6, GraphPad Software, USA) was used for all statistical analyses, and *P* values < 0.05 were considered statistically significant. Contractile responses are expressed as mean percentages of KPSS constriction ± SEM, consistent with previously published data (Greenstein *et al*., 2009; Withers *et al*., 2011), and curves fitted using non‐linear regression analysis. LogEC_50_ values were calculated from individual concentration–response curves fitted using non‐linear regression analysis with a sigmoidal function. Data were analysed using two‐way ANOVA following by Bonferroni *post hoc* test or by Student's unpaired or paired *t*‐test as appropriate.

### Materials

#### Myography

All salts for PSSs, noradrenaline, phenylephrine and carbachol were purchased from Sigma‐Aldrich, Gillingham, Dorset, UK. All other pharmacological tools were purchased from R&D Systems, Abingdon, Oxfordshire, UK.

#### Western blot analysis

General consumables were purchased from Sigma‐Aldrich. Reagents for RIPA buffer were purchased from Roche Diagnostics, Burgess Hill, West Sussex, UK. FastPrep‐24 5G instrument was purchased from MP Biomedicals Inc., Santa Ana, California Antibodies used were as follows: AMPKα (Cell Signaling Technology, Hitchin, Hertfordshire, Cat #2603 RRID: AB_490795), phosphorylated‐AMPKα (Thr^172^) (Cell Signaling Technology, Cat #2535, RRID: AB_331250), β_3_‐adrenoceptor (Novus Biologicals, Abingdon, Oxfordshire, NBP1‐00716, RRID:AB_1502640), eNOS (Santa Cruz Biotechnology, Dallas, Texas, USA, Cat #sc‐654 RRID: AB_631423) and donkey anti‐rabbit HRP (Jackson ImmunoResearch Laboratories, Ely, Cambridgeshire, Cat #711‐035‐152, RRID:AB_10015282). All other Western blot materials were purchased from Bio‐Rad Laboratories.

#### Immunohistochemistry

General consumables were purchased from Sigma‐Aldrich. Antibodies purchased were as follows: K_v_7.4 (Santa Cruz Biotechnology, Cat #sc‐50 417, RRID: AB_2131729), Biotin‐SP conjugated anti‐rabbit secondary antibody (Jackson ImmunoResearch Laboratories, USA, Cat #711‐065‐152, RRID: AB_2340593), vectastain ABC complex and DAB solution (Vector Laboratories, Peterborough, Cambridgeshire, UK, ABC: Cat #PK‐6100, RRID:AB_2336819 DAB: SK‐4100, RRID:AB_2336382).

#### Assays

All general consumables were purchased from Sigma‐Aldrich. The Griess reagent kit was purchased from Promega, Chilworth, Southampton, USA. The cAMP, adiponectin and adipokine assay kits were from R&D systems, Abingdon, Oxfordshire, USA.

### Nomenclature of targets and ligands

Key protein targets and ligands in this article are hyperlinked to corresponding entries in http://www.guidetopharmacology.org, the common portal for data from the IUPHAR/BPS Guide to PHARMACOLOGY (Harding *et al*., 2018), and are permanently achieved in the Concise Guide to PHARMACOLOGY 2017/18 (Alexander *et al*., 2017a, 2017b, 2017c).

## Results

### PVAT produces a transferable anticontractile factor

**Figure 1:**
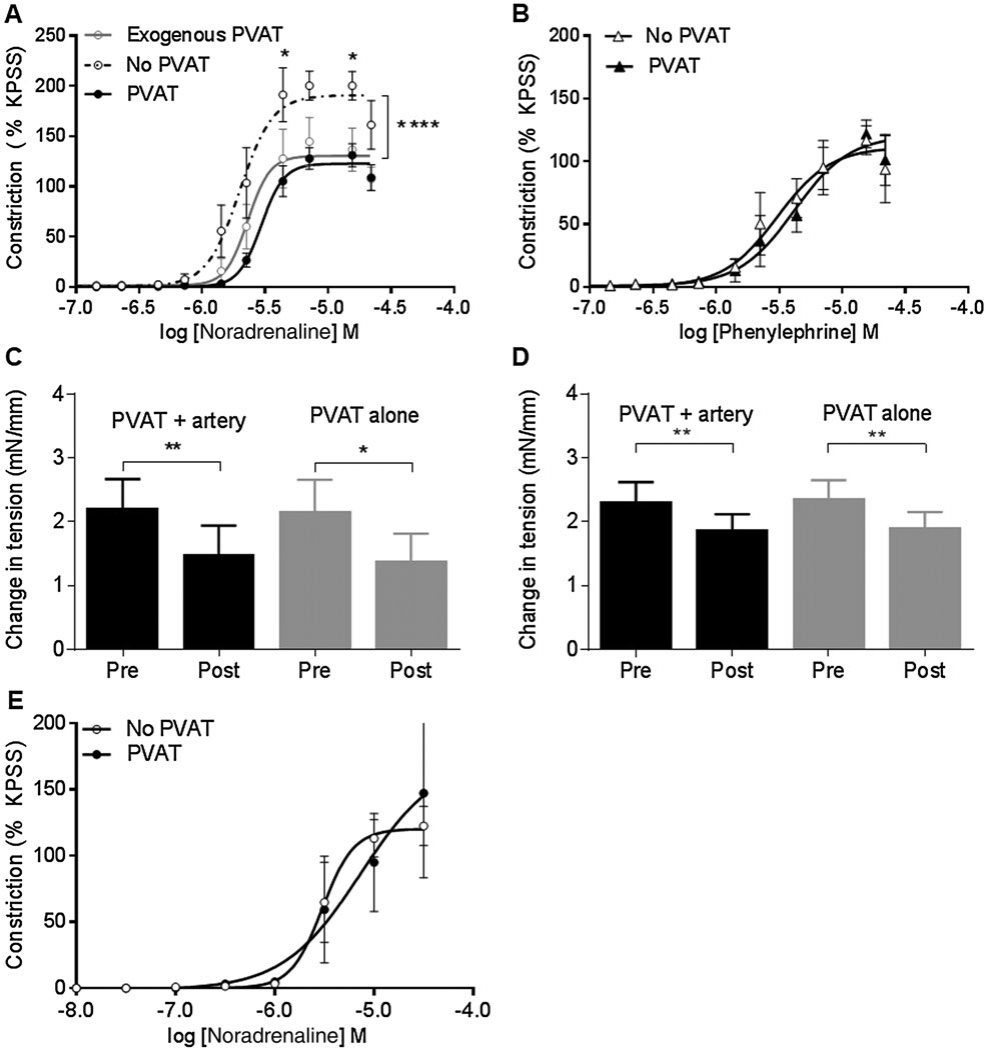
The anticontractile capacity of PVAT is produced by secretion of an endothelium‐independent soluble factor. The presence of PVAT reduced the vasoconstrictor response to (A) noradrenaline (*n* = 18). The contractile response to noradrenaline was also reduced in vessels with exogenous PVAT (no PVAT vs. exogenous PVAT: *P* < 0.05, PVAT vs. exogenous PVAT: *P* = NS, *n* = 5 and 6 respectively). The presence of PVAT had no effect on the vasoconstrictor response to (B) phenylephrine (PVAT *n* = 10, no PVAT *n* = 12). Both donor PVAT and recipient arteries were stimulated with 30 μmol·L^−1^ noradrenaline prior to transfer. (C) Transfer of solution from donor PVAT to a recipient artery lacking PVAT produced a significant reduction in tension both in the presence (*n* = 6) and absence (*n* = 6) of an artery with the PVAT. (D) Transfer of solution from donor PVAT incubated in calcium‐free PSS to a recipient artery produced a significant reduction in tension in the presence and absence of PVAT (*n* = 9) and in the absence of an artery with the PVAT (*n* = 8). The total external calcium concentration of the recipient bath was kept constant at 1.6 mmol·L^−1^ after transfer. (E) The anticontractile effect is absent in endothelium‐denuded arteries (*P* = NS, *n* = 6) Data are expressed as mean ± SEM. *P < 0.05, two‐way ANOVA or paired *t*‐test.

In endothelium‐intact segments of rat mesenteric small arteries, the vasoconstrictor response to noradrenaline (Figure 1A) but not phenylephrine was reduced in the presence of PVAT (Figure 1B). The vasoconstrictor response to noradrenaline was reduced in vessels lacking PVAT with exogenous PVAT suspended in the bath (Figure 1A), suggesting that the anticontractile effect was due to secretion of a soluble factor.

Transfer of solutions from donor PVAT with and without an artery stimulated with noradrenaline to a recipient artery lacking PVAT pre‐contracted with noradrenaline produced a significant reduction in tension (Figure 1C), confirming that the anticontractile effect was due to secretion of a soluble factor. Also, relaxation occurred following incubation of donor PVAT with and without an artery in calcium‐free external PSS solution and transfer to a recipient artery, indicating that the anticontractile effect is calcium‐independent (Figure 1D).

In endothelium‐denuded arteries, the vasoconstrictor response to noradrenaline was not reduced by the presence of PVAT (Figure 1E), confirming that the anticontractile factor is endothelium‐independent.

### Adipocyte located β_3_‐adrenergic activation produces a PVAT‐derived anticontractile effect via Gα_s_ signalling

**Figure 2:**
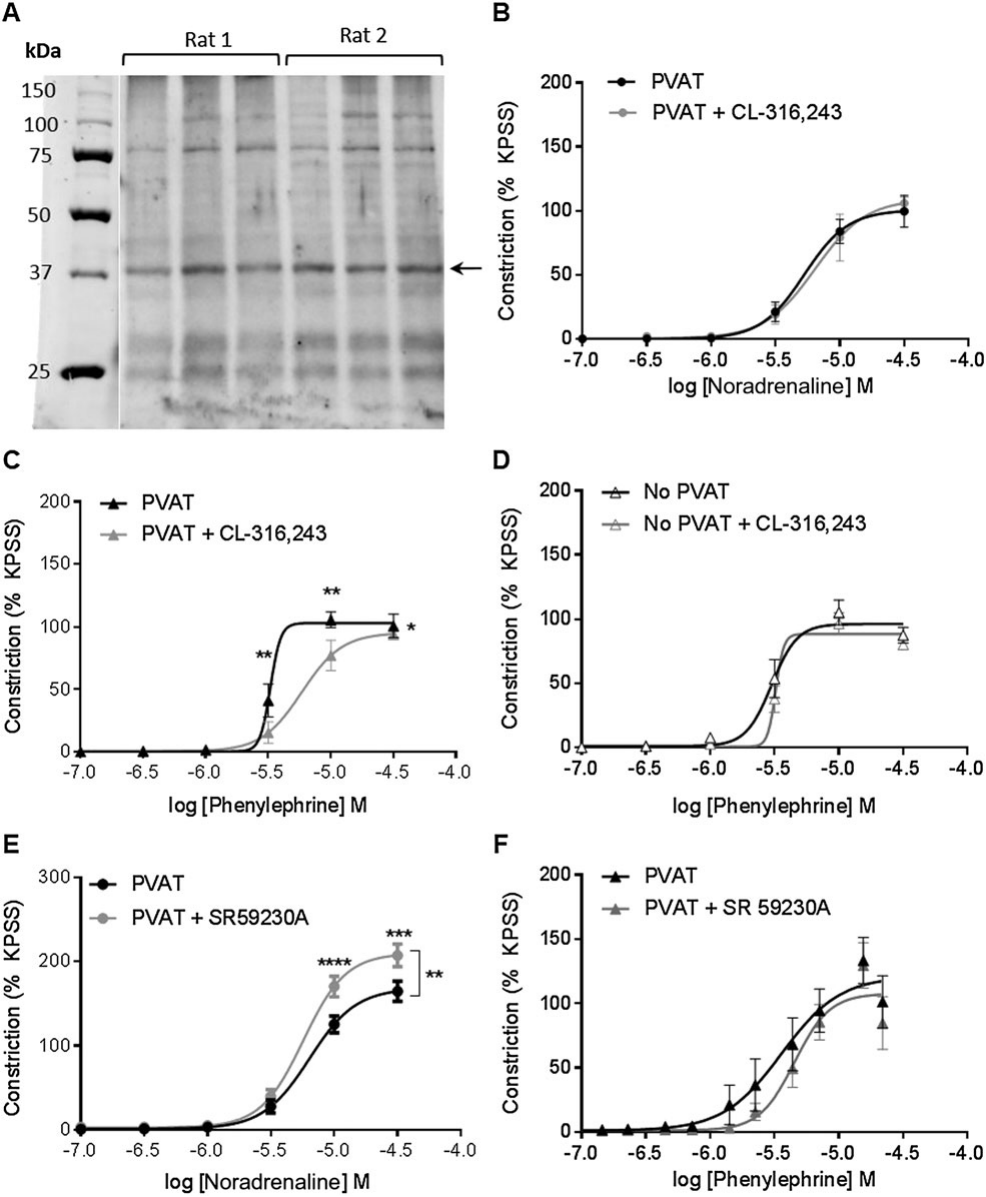
β_3_‐adrenoceptor activation contributes to the PVAT anticontractile effect. (A) Representative Western blot indicating expression of β_3_‐adrenoceptors within rat mesenteric PVAT. Three independent samples were analysed from each rat on one membrane. Incubation of intact vessels with PVAT with 10 μmol·L^−1^ CL‐316243 for 30 min did not alter the response to (B) noradrenaline (*n* = 5) but enhanced the anticontractile effect of (C) phenylephrine (*n* = 5). (D) Incubation of vessels with endothelium but lacking PVAT with 10 μmol·L^−1^ CL‐316243 did not alter the contractile response to phenylephrine (*n* = 5). Incubation of mesenteric vessels with PVAT with the β_3_‐adrenoceptor antagonist, 1 μmol·L^−1^ SR 59230A for 30 min potentiated constriction to (E) noradrenaline (*n* = 7) but had no effect on the contractile response to (F) phenylephrine (*P* = NS, *n* = 7). Data are expressed as mean ± SEM. *P < 0.05, two‐way ANOVA.

Western blot analysis indicated the presence of β_3_‐adrenoceptors within the PVAT of rat mesenteric arteries (Figure 2A); therefore, we investigated the contribution of β_3_‐adrenoceptor‐mediated signalling to the anticontractile effect. The presence of CL‐316243 had no effect on vasoconstrictor response to noradrenaline (Figure 2B) but reduced the effect of phenylephrine (Figure 2C), indicating a role for β_3_‐adrenoceptor activation in mediating the anticontractile effect of noradrenaline on PVAT. Incubation of vessels with intact endothelium but lacking PVAT with CL‐316243 had no effect on the vasoconstrictor response to either of the agonists tested (Figure 2D) confirming that the changes observed are a consequence of β_3_‐adrenoceptor activation within PVAT. Moreover, the β_3_‐adrenoceptor antagonist, SR 59230A, increased the contractile response to noradrenaline in vessels with PVAT (Figure 2E) but had no effect on the response to phenylephrine (Figure 2F).

**Figure 3:**
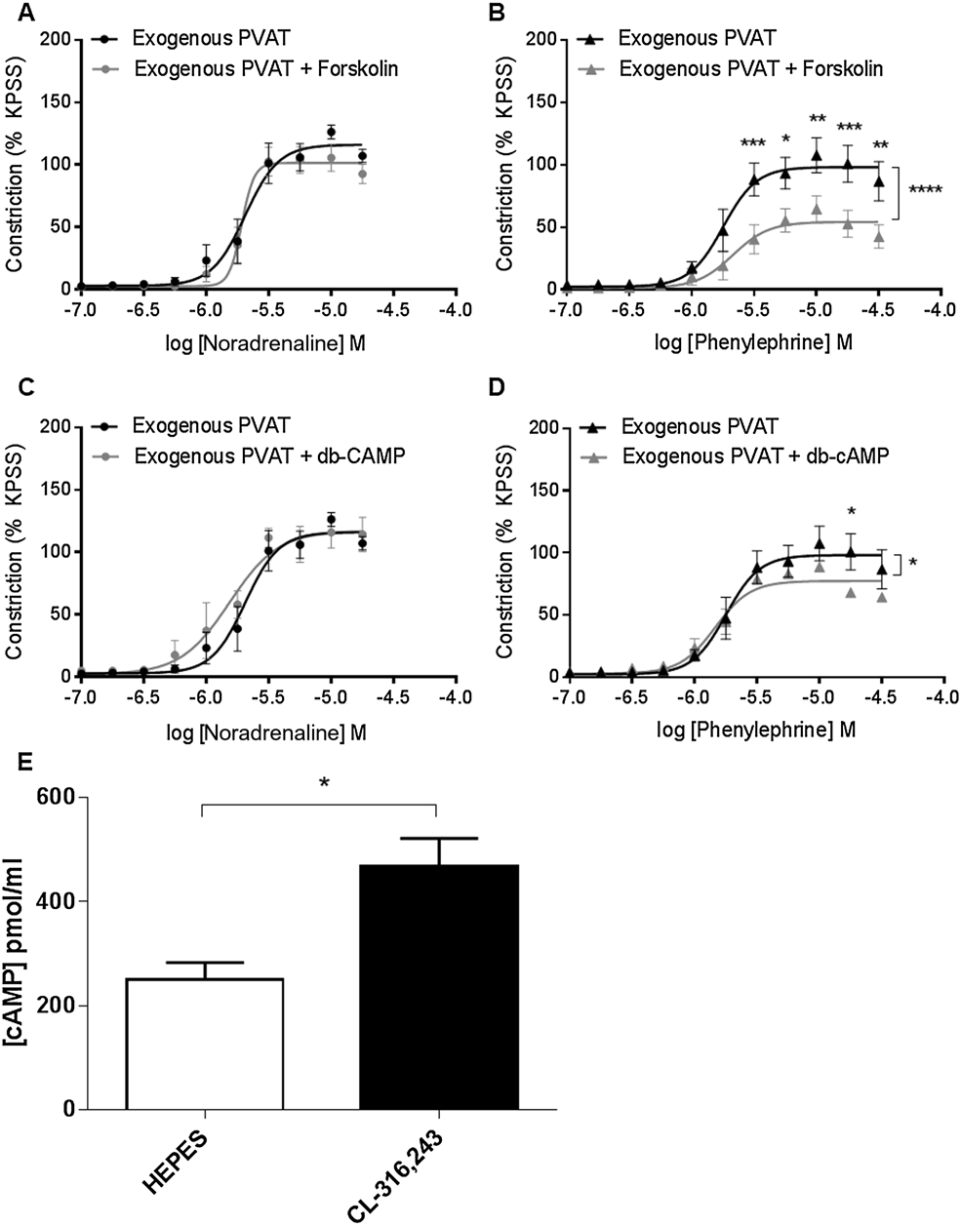
Gαs signalling contributes to the noradrenaline‐induced PVAT anticontractile effect. Incubation of exogenous PVAT with 1 μmol·L^−1^ forskolin for 30 min prior to placement within the myograph bath had no effect on the vasoconstrictor response to (A) noradrenaline (*n* = 6) but reduced the contractile response to (B) phenylephrine (*n* = 6). Incubation of vessels with PVAT with the cAMP analogue, 10 μmol·L^−1^ db‐cAMP had no effect on constriction to (C) noradrenaline (*n* = 6) but reduced the vasoconstrictor response to (D) phenylephrine (*n* = 6). (E) Intracellular cAMP levels increased following stimulation with 10 μmol·L^−1^ CL‐316243 (*P* < 0.05, *n* = 6). Data are expressed as mean ± SEM. *P < 0.05, two‐way ANOVA.

Incubation of exogenous PVAT with forskolin had no effect on the vasoconstrictor response to noradrenaline (Figure 3A) but reduced the effect of phenylephrine (Figure 3B). Incubation with db‐cAMP had no effect on the vasoconstrictor response to noradrenaline (Figure 3C) but reduced the vasoconstrictor response to phenylephrine (Figure 3D). Intracellular cAMP levels were increased from 250 ± 33 to 467.4 ± 54 pmol·mL^−1^ within PVAT following stimulation with CL‐316243 indicating that β_3_‐adrenoceptor stimulation involves Gα_s_ signalling (Figure 3E).

### K_v_7 channel activation contributes to noradrenaline‐mediated anticontractile capacity

**Figure 4:**
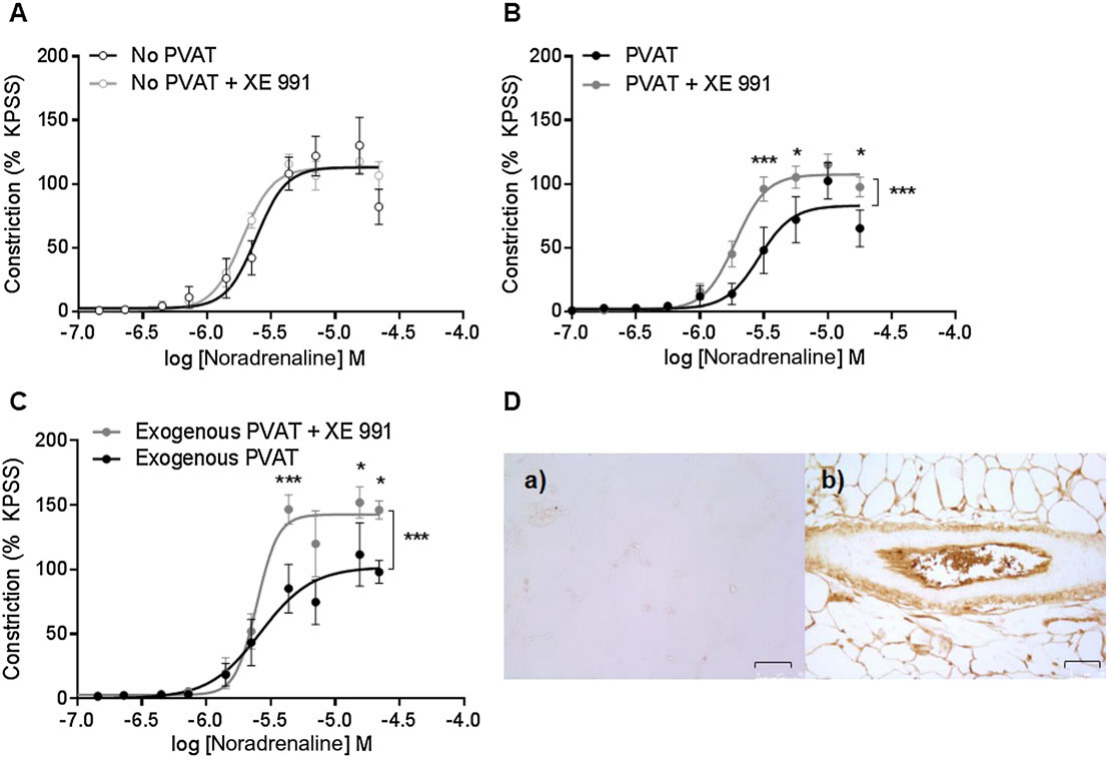
K_v_7 channel inhibition prevents PVAT anticontractile effect. (A) Incubation of vessels lacking PVAT with the K_v_7 inhibitor, XE 991 (10 μmol^.^L^‐1^) for 30 min had no effect on the contractile response to noradrenaline (*n* = 6) but (B) potentiated the contractile response to noradrenaline in vessels with PVAT (*n* = 6). (C) Incubation of exogenous PVAT with XE 991 for 30 min prior to addition to the myograph bath produced an increase in the contractile response in vessels lacking PVAT compared to vessels with control exogenous PVAT (*n* = 5). (D) Immunostaining showed no staining when mesenteric bed samples were incubated with rabbit IgG (a) but clear staining within the PVAT, endothelial layer and adventitial layer of the mesenteric artery when incubated with anti‐K_v_7.4 antibody (b). Images were obtained at 20x magnification and scale bar represents 75 μm. Data are expressed as mean ± SEM. * *P* < 0.05, one‐way ANOVA.

Incubation with the K_v_7 inhibitor, XE 991, did not alter the vasoconstrictor response to noradrenaline in vessels lacking PVAT (Figure 4A) but potentiated the response to noradrenaline in vessels with PVAT (Figure 4B). Incubation of exogenous PVAT with XE 991 prior to placement within the myograph bath reversed the anticontractile effect of PVAT (Figure 4C), indicating that K_v_7 channel activation *within* PVAT contributes to the noradrenaline‐induced anticontractile capacity. Immunostaining for K_v_7.4 shows K_v_7.4 expression within PVAT, the endothelial layer and the adventital layer of the mesenteric artery (Figure 4D).

### NO produced by adipocytes plays a key role in the anticontractile effect

**Figure 5:**
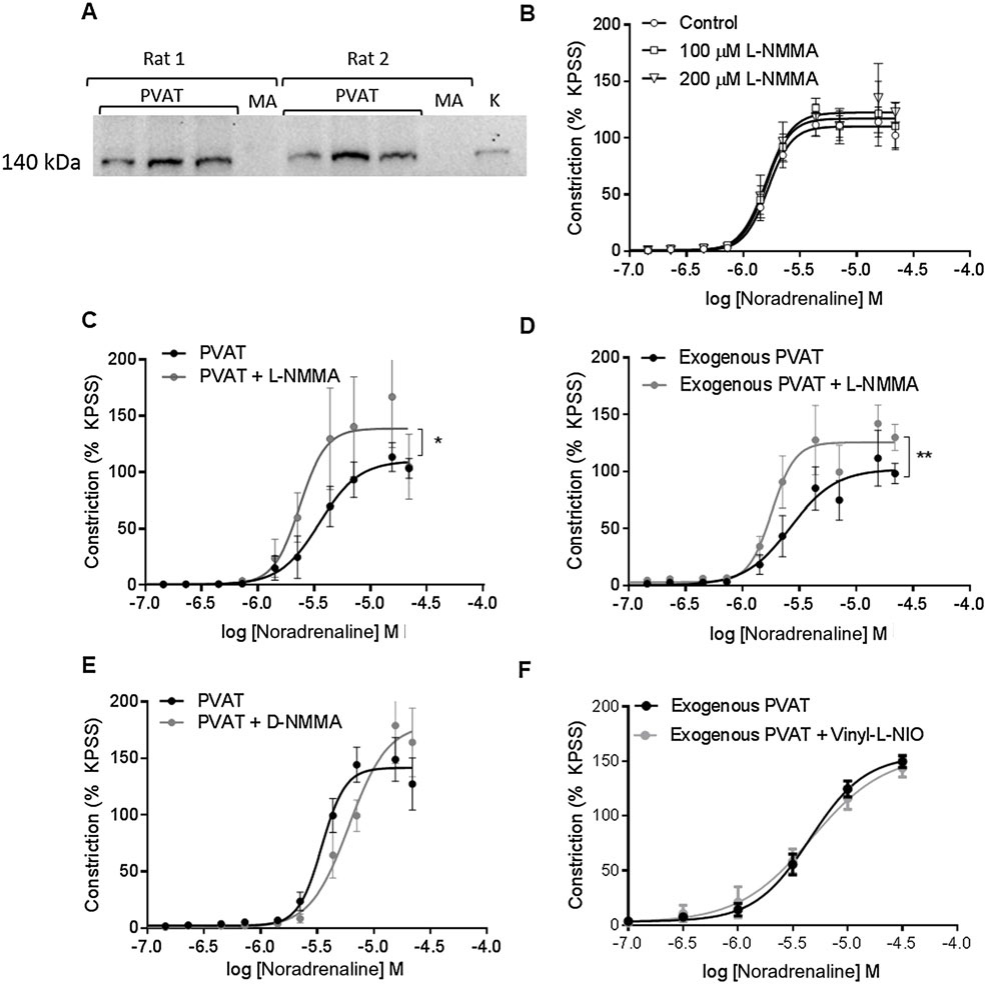
PVAT‐derived NO plays a key role. (A) Representative Western blot showing expression of eNOS within PVAT and mesenteric artery (MA). Kidney (K) was used as a positive control. (B) Incubation of vessels with endothelium but lacking PVAT with either 100 μmol.L‐1 or 200 μmol.L‐1 L‐NMMA had no effect on the contractile response to noradrenaline (n = 5). (C) Incubation with 100 μmol.L‐1 L‐NMMA increased the vasoconstrictor response to noradrenaline in vessels with intact PVAT (n = 6). (D) Incubation of exogenous PVAT with μmol.L‐1 L‐NMMA for 30 min prior to suspension within the myograph bath produced an increase in the contractile response in vessels lacking PVAT compared to vessels with control exogenous PVAT (n = 5). (E) Incubation of vessels with PVAT with D‐NMMA did not alter the contractile response to noradrenaline (n = 5). (F) Incubation of exogenous PVAT with 100 nmol.L‐1 Vinyl‐L‐NIO for 30 min prior to suspension within the myograpth bath had no effect on contractile response (n = 8). Data are expressed mean ± SEM. * P < 0.05, two‐way ANOVA.

**Figure 6:**
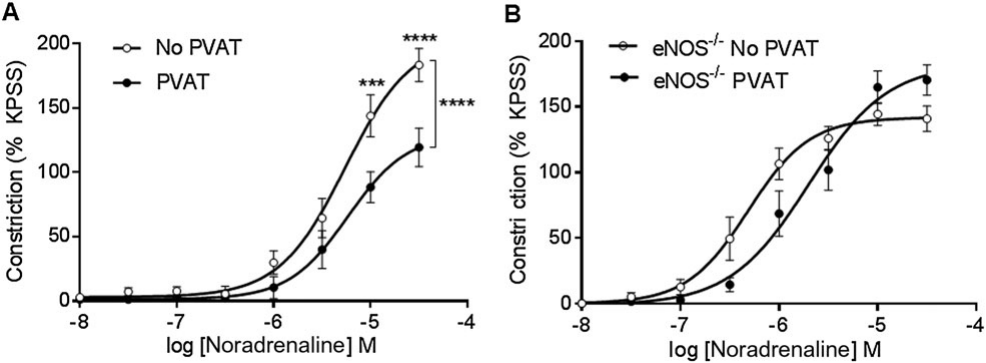
The PVAT anticontractile effect is lost in eNOS^−/−^ mice. (A) In C57BL6/j mice, the vasoconstrictor response to noradrenaline is reduced in the presence of PVAT (*n* = 8). (B) The presence of PVAT had no effect on the vasoconstrictor response to noradrenaline in mesenteric arteries from eNOS^−/−^ mice (*n* = 8). Data are expressed as mean ± SEM. *P < 0.05, two‐way ANOVA.

Western blot analysis demonstrated higher eNOS expression within PVAT than the mesenteric artery (Figure 5A). Incubation with the NOS inhibitor, l‐NMMA, did not alter the vasoconstrictor response to noradrenaline in vessels with intact endothelium but lacking PVAT (Figure 5B), but the vasoconstrictor response was increased in the presence of PVAT (Figure 5C). Incubation of exogenous PVAT with l‐NMMA prior to placement within the myograph bath increased the vasoconstrictor response to noradrenaline (Figure 5D), indicating that PVAT‐derived NO may contribute to the noradrenaline‐induced anticontractile capacity. Incubation with d‐NMMA, an inactive enantiomer of l‐NMMA, had no effect on the contractile response of vessels with PVAT (Figure 5E). Incubation of exogenous PVAT with nNOS inhibitor vinyl‐l‐NIO had no effect on the contractile response (Figure 5F), indicating that eNOS may be the NOS isoform responsible for NO release from PVAT. Moreover, the anticontractile effect of PVAT on the vasoconstrictor response to noradrenaline present in C57BL6/j mice was absent in eNOS^−/−^, mice indicating that eNOS activation contributes to the release of NO from PVAT (Figure 6).

### β_3_‐Adrenoceptor activation within PVAT does not alter adiponectin release

**Figure 7:**
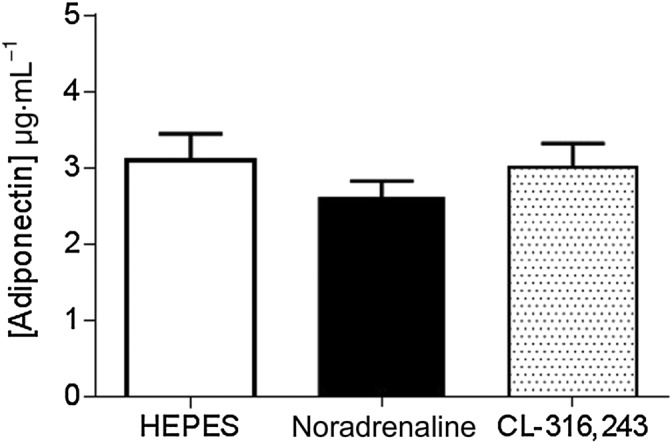
Total adiponectin secretion from PVAT is not altered by β_3_‐adrenoceptor activation. Total adiponectin released from PVAT into surrounding solution was not increased by adrenergic stimulation (*n* = 6).

Abundant amounts of adiponectin were found to be basally secreted from PVAT; however, this was unaltered following stimulation with either noradrenaline or CL‐316243 (Figure 7).

## Discussion

The present study investigated the mechanisms of the noradrenaline‐induced PVAT anticontractile effect. The main findings were that (i) β_3_‐adrenoceptor‐mediated signalling contributes to the PVAT anticontractile effect; (ii) K_v_7 channel activation *within* PVAT contributes to release of a PDRF; and (iii) PVAT‐derived NO plays a key role in the noradrenaline‐induced PVAT anticontractile effect.

Consistent with previously published data (Briones *et al*., 2005; Garland *et al*., 2011; Flacco *et al*., 2013), we demonstrated no functional role of β_3_‐adrenoceptor‐mediated signalling in the contractile response to noradrenaline in vessels lacking PVAT. This suggests that the effects observed in the present study were a consequence of β_3_‐adrenoceptor activation within the PVAT. This is supported by our previous observations of β_3_‐adrenoceptor expression within mouse mesenteric PVAT and the effects of adipocyte β_3_‐adrenoceptor inhibition on vessel tension (Saxton *et al*., 2018). In this study, we report a similar β_3_‐adrenoceptor‐mediated mechanism in rat PVAT and have for the first time identified PVAT‐derived NO as a relaxing factor released upon β_3_‐adrenoceptor activation.

Adipocyte β_3_‐adrenoceptors are likely activated by sympathetically derived NA. The role of β_3_‐adrenoceptors in catecholamine induced lipolysis has been well characterized (Bartness *et al*., 2014; Rebuffe‐Scrive, 1991; Robidoux *et al*., 2006), and the origin of sympathetic fibres in epididymal and inguinal fat depots has been extensively studied using viral transneuronal tract tracers (Bamshad *et al*., 1998; Song *et al*., 2009; Nguyen *et al*., 2014). Denervation of these fat depots will lead to a decrease in lipolysis (Rooks *et al*., 2005; Foster and Bartness, 2006); however, until recently, the importance of sympathetic nerves in PVAT was unknown. Previously, using immunohistochemistry, we have confirmed the presence of sympathetic nerves in mouse mesenteric PVAT (Saxton *et al*., 2018). In addition, we confirmed a functional role for sympathetic nerves in PVAT, as pharmacological sympathetic denervation of PVAT abolished the anticontractile effect. Therefore, it is sympathetically derived NA which activates β_3_‐adrenoceptors, triggering the release of a relaxing factor.

Dual coupling of β_3_‐adrenoceptors to both Gα_s_ and Gα_i_ proteins has been reported in 3T3‐F440A adipocytes (Soeder *et al*., 1999). We highlight an important role of the β_3_‐adrenoceptor/Gα_s_/cAMP signalling pathway in the PVAT anticontractile capacity as β_3_‐adrenoceptor stimulation resulted in cAMP production and incubation with forskolin or db‐cAMP was able to mimic the PVAT anticontractile effect. This is supported by previous studies showing that β_3_‐adrenoceptors within cultured adipocytes have a tendency towards Gα_s_ protein coupling following stimulation with physiologically relevant catecholamine concentrations (Soeder *et al*., 1999; Robidoux *et al*., 2006).

Our finding that K_v_7 channel inhibition does not alter the contractile response to noradrenaline in vessels lacking PVAT was surprising given the key role of K_v_7 channels in the regulation of vascular tone (Ng *et al*., 2011; Chadha *et al*., 2012). The majority of studies investigating the effect of K_v_7 channel inhibition on vascular tone has reported increased myogenicity in the presence of XE 991 rather than investigating its effect on the arterial response to vasoconstrictor agonists or have demonstrated activation/inhibition of currents using single‐cell electrophysiology in the absence of spasmogens (Ng *et al*., 2011; Chadha *et al*., 2012). Moreover, we found very little K_v_7.4 expression within the arterial medial layer consistent with the absence of a functional role, and our data are supported by an earlier myograph study using rat mesenteric artery (Li *et al*., 2013).

Substantial evidence suggests a role for voltage‐gated K^+^ channels, especially K_v_7 in mediating the PVAT anticontractile effect in resistance arteries (Verlohren *et al*., 2004; Schleifenbaum *et al*., 2010; Zavaritskaya *et al*., 2013). For the first time, we have shown K_v_7.4 expression within PVAT and that specific inhibition of K_v_7 within PVAT reversed the noradrenaline‐induced anticontractile effect in health. This suggests a crucial role for activation of K_v_7 located *within* PVAT in mediating the release of a relaxing factor in health. These findings contradict previous studies that suggested a role for vascular smooth muscle K_v_7 channels in mediating the anticontractile effect (Fang *et al*., 2009; Schleifenbaum *et al*., 2010; Zavaritskaya *et al*., 2013). However, our experimental design allowed specific inhibition of K_v_7 within PVAT without affecting smooth muscle channels enabling identification of the tissue location of the channels involved in the PVAT anticontractile effect. Moreover, previous studies did not investigate K_v_7 expression within PVAT, and on the basis of their results, a role for PVAT K_v_7 activation could not be excluded.

Our data showing a crucial role for K_v_7 within the adipocytes are supported by observations of voltage‐gated K^+^ channels within rat white adipocytes (Ramirez‐Ponce *et al*., 1996) that can be modulated by noradrenaline and cAMP (Ramirez‐Ponce *et al*., 1998). Moreover, evidence has emerged that K_v_7 channel activation contributes to the vasodilator response induced by β‐adrenoceptor agonists through a mechanism involving cAMP and http://www.guidetopharmacology.org/GRAC/FamilyDisplayForward?familyId=284, because K_v_7 channel inhibitor linopirdine was able to inhibit relaxations induced by isoprenaline and forskolin (Chadha *et al*., 2012), and phosphorylation of K_v_7.4 by PKA has been reported to shift the activation curve leading to enhanced K_v_7.4 activity (Chambard and Ashmore, 2005). Taken together with the observed increased cAMP levels, we suggest that β_3_‐adrenoceptor stimulation within PVAT and subsequent cAMP production leads to K_v_7 activation. This leads to the movement of potassium out of the adipocyte down its electrochemical gradient and hyperpolarization that drives calcium entry with subsequent activation of downstream signalling pathways leading to the release of NO.

Consistent with earlier work, we were able to show that PVAT releases a transferable factor (Lohn *et al*., 2002; Greenstein *et al*., 2009; Lynch *et al*., 2013), and we demonstrate that this factor is adipocyte‐derived NO as the anticontractile effect reversed in the presence of specific PVAT NOS inhibition and stimulation with CL‐316243, but not phenylephrine increased NO release from PVAT. Consistent with previous studies, the effects of this factor are endothelium‐independent (Greenstein *et al*., 2009; Saxton *et al*., 2018).

Several studies have demonstrated a link between β_3_‐adrenoceptor‐mediated signalling and NO production by adipocytes (Canova *et al*., 2006; Hodis *et al*., 2011), and recent electrophysiological studies in non‐contracted vessels demonstrated that β_3_‐adrenoceptor‐mediated stimulation of PVAT produced myocyte hyperpolarization that could be partially reversed by the presence of l‐NMMA (Weston *et al*., 2013). Moreover, our data support previous findings in human s.c. arteries (Greenstein *et al*., 2009) and mouse mesenteric arteries (Lynch *et al*., 2013) where NOS inhibition was reported to attenuate PVAT function, and we have recently shown a crucial role for activation of PKG, a downstream signalling component in the NO pathway, in the noradrenaline‐induced anticontractile effect in mice (Withers *et al*., 2014).

l‐NMMA inhibits all three isoforms of NOS (eNOS, nNOS and iNOS) but may also have non‐specific effects. However, the lack of effects of the inactive enantiomer, d‐NMMA, and the specific nNOS inhibitor, vinyl‐l‐NIO, strongly suggested that the observed effects of l‐NMMA in rat PVAT were a consequence of eNOS inhibition. Endothelial NOS (eNOS) plays a key role in white adipose tissue metabolism (Sansbury *et al*., 2012) and is activated by increased intracellular calcium (Busse and Mulsch, 1990). Moreover, the PVAT anticontractile effect was absent in eNOS^−/−^ mice, and we were able to detect greater eNOS expression in PVAT than mesenteric artery suggesting that it is the key NOS isoform catalyzing NO production within PVAT.

Adiponectin promotes NO production within the endothelium (Cheng *et al*., 2007), and previous studies within our laboratory have suggested a key role for adiponectin as a relaxing factor mediating the anticontractile effect in human s.c. (Greenstein *et al*., 2009) and mouse mesenteric (Lynch *et al*., 2013) arteries and in response to β_3_‐adrenoceptor‐mediated stimulation of non‐contracted rat mesenteric arteries (Weston *et al*., 2013). Also, serum levels of adiponectin were increased in rats following 7 day infusion of CL‐316243 (Zhang *et al*., 2002), and adiponectin secretion from mouse mesenteric PVAT is significantly reduced when incubated with SR 59230A (Saxton *et al*., 2018). Previously, adiponectin has been shown to induce vasorelaxation in rat (Greenstein *et al*., 2009) and mouse (Lynch *et al*., 2013) mesenteric arteries and myocyte hyperpolarization in non‐contracted vessels (Weston *et al*., 2013). We observed high basal adiponectin secretion from PVAT; however, no changes in total adiponectin secretion were observed following stimulation with noradrenaline or CL‐316243. This may be because biologically relevant changes in the ratio of high MW : low MW isoforms could not be detected using commercially available ELISA kits (Brochu‐Gaudreau *et al*., 2010). AMPK is a major downstream target of adiponectin (Yamauchi *et al*., 2007), and we show AMPK phosphorylation in unstimulated PVAT was not altered by β‐adrenoceptor‐mediated stimulation, suggesting that adiponectin may be constitutively released from PVAT and could facilitate NO production by the adipocytes in response to stimulation of β‐adrenoceptors.

**Figure 8:**
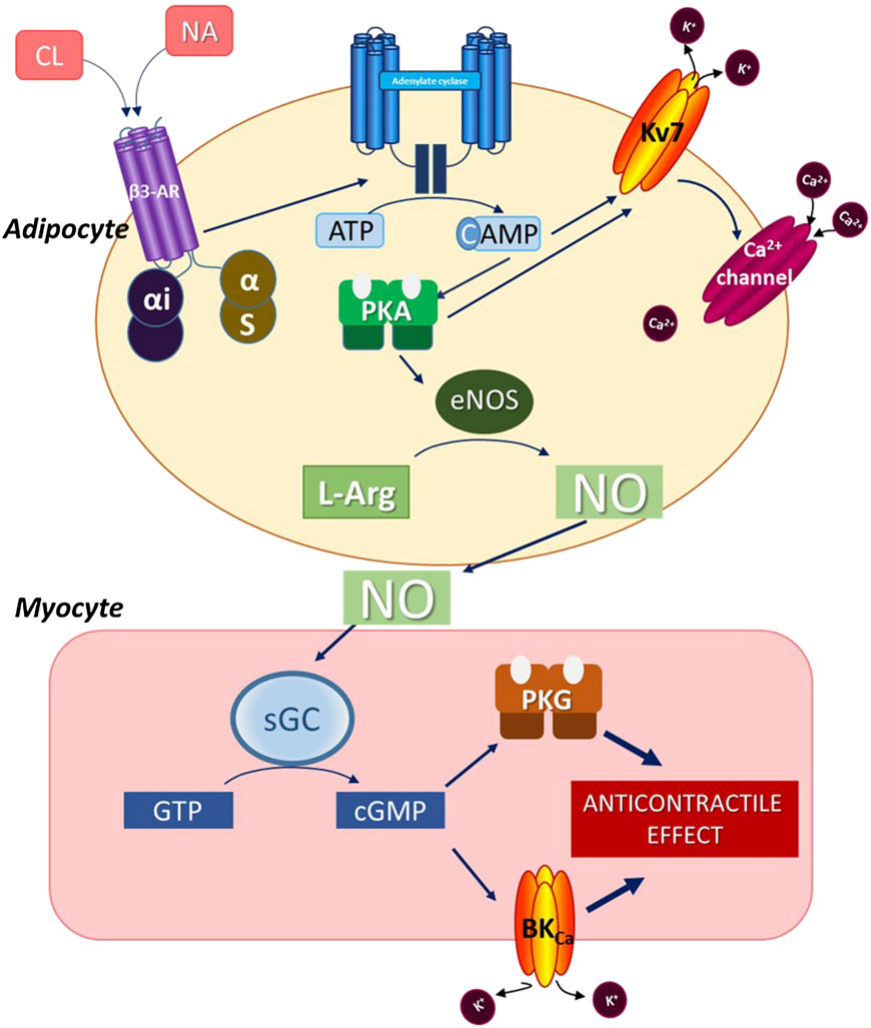
Proposed pathway for nitric oxide release by PVAT and how it results in the PVAT anticontractile effect. Noradrenaline (NA) activates β_3_‐adrenoceptors within PVAT leading to increased cAMP production. This activates K_v_7 within PVAT leading to an efflux of potassium and subsequent hyperpolarisation that drives calcium entry into the adipocyte and activation of endothelial nitric oxide synthase (eNOS) which catalyses the production of NO from L‐arginine (L‐Arg). NO is released from PVAT and acts on vascular smooth muscle cells leading to hyperpolarisation and vasodilatation which opposes vasoconstriction resulting in an anticontractile effect.

The results presented show that adipocyte β_3_‐adrenoceptor stimulation leads to increased intracellular cAMP production and KCNQ activation via increased intracellular calcium leading to release of relaxing factors, one of which is NO (Figure 7). For the first time, we demonstrate that noradrenaline stimulates release of adipocyte‐derived NO. This NO is a key PDRF and modulates arterial contraction (Figure 8). These findings support the targeting of NOS for the treatment of PVAT dysfunction associated with obesity and the metabolic syndrome.

## Author contributions

C.E.B., S.B.W., S.N.S. and A.M.H. designed the experiments and wrote the paper. C.E.B., S.B.W., S.N.S., N.B. and R.G.A. performed the experiments and analysed the data. All authors approved the final version of the paper. All experiments were performed in the Heagerty laboratory, University of Manchester.

## Conflicts of interest

The authors declare no conflicts of interest.

## Declaration of transparency and scientific rigour

This http://onlinelibrary.wiley.com/doi/10.1111/bph.13405/abstract acknowledges that this paper adheres to the principles for transparent reporting and scientific rigour of preclinical research recommended by funding agencies, publishers and other organisations engaged with supporting research.

## References

[bph14433-bib-0001] Aghamohammadzadeh R , Greenstein AS , Yadav R , Jeziorska M , Hama S , Soltani F *et al* (2013). Effects of bariatric surgery on human small artery function: evidence for reduction in perivascular adipocyte inflammation, and the restoration of normal anticontractile activity despite persistent obesity. J Am Coll Cardiol 62: 128–135. 23665100 10.1016/j.jacc.2013.04.027 PMC3791397

[bph14433-bib-0002] Alexander SPH , Striessnig J , Kelly E , Marrion NV , Peters JA , Faccenda E *et al* (2017a). The Concise Guide to PHARMACOLOGY 2017/18: Voltage‐gated ion channels. Br J Pharmacol. 174 (Suppl. 1): S160–S194. 29055033 10.1111/bph.13884 PMC5650668

[bph14433-bib-0003] Alexander SPH , Fabbro D , Kelly E , Marrion NV , Peters JA , Faccenda E *et al* (2017b). The Concise Guide to PHARMACOLOGY 2017/18: Enzymes. Br J Pharmacol. 174 (Suppl. 1): S272–S359. 29055034 10.1111/bph.13877 PMC5650666

[bph14433-bib-0004] Alexander SPH , Christopoulos A , Davenport AP , Kelly E , Marrion NV , Peters JA *et al* (2017c). The Concise Guide to PHARMACOLOGY 2017/18: G protein‐coupled receptors. Br J Pharmacol. 174 (Suppl. 1): S17–S129. 29055040 10.1111/bph.13878 PMC5650667

[bph14433-bib-0005] Babu BR , Griffith OW (1998). N5‐(1‐Imino‐3‐butenyl)‐L‐ornithine. A neuronal isoform selective mechanism‐based inactivator of nitric oxide synthase. J Biol Chem 273: 8882–8889. 9535869 10.1074/jbc.273.15.8882

[bph14433-bib-0006] Bamshad M , Aoki VT , Adkison MG , Warren WS , Bartness TJ (1998). Central nervous system origins of the sympathetic nervous system outflow to white adipose tissue. Am J Physiol 275: R291–R299. 9688991 10.1152/ajpregu.1998.275.1.R291

[bph14433-bib-0007] Bartness TJ , Liu Y , Shrestha YB , Ryu V (2014). Neural innervation of white adipose tissue and the control of lipolysis. Front Neuroendocrinol 35: 473–493. 24736043 10.1016/j.yfrne.2014.04.001 PMC4175185

[bph14433-bib-0009] Briones AM , Daly CJ , Jimenez‐Altayo F , Martinez‐Revelles S , Gonzalez JM , McGrath JC *et al* (2005). Direct demonstration of β1‐ and evidence against β2‐ and β3‐adrenoceptors, in smooth muscle cells of rat small mesenteric arteries. Br J Pharmacol 146: 679–691. 16113691 10.1038/sj.bjp.0706369 PMC1751207

[bph14433-bib-0010] Brochu‐Gaudreau K , Rehfeldt C , Blouin R , Bordignon V , Murphy BD , Palin MF (2010). Adiponectin action from head to toe. Endocrine 37: 11–32. 20963555 10.1007/s12020-009-9278-8

[bph14433-bib-0011] Busse R , Mulsch A (1990). Calcium‐dependent nitric‐oxide synthesis in endothelial cytosol is mediated by calmodulin. FEBS Lett 265: 133–136. 1694782 10.1016/0014-5793(90)80902-u

[bph14433-bib-0012] Canova NK , Lincova D , Kmonickova E , Kamenikova L , Farghali H (2006). Nitric oxide production from rat adipocytes is modulated by β(3)‐adrenergic receptor agonists and is involved in a cyclic AMP‐dependent lipolysis in adipocytes. Nitric Oxide‐Biol Ch 14: 200–211. 10.1016/j.niox.2005.06.006 16413212

[bph14433-bib-0013] Chadha PS , Zunke F , Davis AJ , Jepps TA , Linders JT , Schwake M *et al* (2012). Pharmacological dissection of K(v)7.1 channels in systemic and pulmonary arteries. Br J Pharmacol 166: 1377–1387. 22251082 10.1111/j.1476-5381.2012.01863.x PMC3417453

[bph14433-bib-0014] Chambard JM , Ashmore JF (2005). Regulation of the voltage‐gated potassium channel KCNQ4 in the auditory pathway. Pflugers Arch 450: 34–44. 15660259 10.1007/s00424-004-1366-2

[bph14433-bib-0015] Cheng KK , Lam KS , Wang Y , Huang Y , Carling D , Wu D *et al* (2007). Adiponectin‐induced endothelial nitric oxide synthase activation and nitric oxide production are mediated by APPL1 in endothelial cells. Diabetes 56: 1387–1394. 17287464 10.2337/db06-1580

[bph14433-bib-0016] Curtis MJ , Alexander S , Cirino G , Docherty JR , George CH , Giembycz MA *et al* (2018). Experimental design and analysis and their reporting II: updated and simplified guidance for authors and peer reviewers. Br J Pharmacol 175: 987–993. 29520785 10.1111/bph.14153 PMC5843711

[bph14433-bib-0017] Fang L , Zhao J , Chen Y , Ma T , Xu G , Tang C *et al* (2009). Hydrogen sulfide derived from periadventitial adipose tissue is a vasodilator. J Hypertens 27: 2174–2185. 19644389 10.1097/HJH.0b013e328330a900

[bph14433-bib-0018] Ferguson RE , Carroll HP , Harris A , Maher ER , Selby PJ , Banks RE (2005). Housekeeping proteins: a preliminary study illustrating some limitations as useful references in protein expression studies. Proteomics 5: 566–571. 15627964 10.1002/pmic.200400941

[bph14433-bib-0019] Flacco N , Segura V , Perez‐Aso M , Estrada S , Seller JF , Jimenez‐Altayo F *et al* (2013). Different β‐adrenoceptor subtypes coupling to cAMP or NO/cGMP pathways: implications in the relaxant response of rat conductance and resistance vessels. Br J Pharmacol 169: 413–425. 23373597 10.1111/bph.12121 PMC3651666

[bph14433-bib-0020] Foster MT , Bartness TJ (2006). Sympathetic but not sensory denervation stimulates white adipocyte proliferation. Am J Physiol Regul Integr Comp Physiol 291: R1,630–R1,637. 16887921 10.1152/ajpregu.00197.2006

[bph14433-bib-0021] Fésüs G , Dubrovska G , Gorzelniak K , Kluge R , Huang Y , Luft FC *et al* (2007). Adiponectin is a novel humoral vasodilator. Cardiovasc Res 75: 719–727. 17617391 10.1016/j.cardiores.2007.05.025

[bph14433-bib-0022] Gao YJ , Lu C , Su LY , Sharma AM , Lee RM (2007). Modulation of vascular function by perivascular adipose tissue: the role of endothelium and hydrogen peroxide. Br J Pharmacol 151: 323–331. 17384669 10.1038/sj.bjp.0707228 PMC2013985

[bph14433-bib-0023] Gao YJ , Zeng ZH , Teoh K , Sharma AM , Abouzahr L , Cybulsky I *et al* (2005). Perivascular adipose tissue modulates vascular function in the human internal thoracic artery. J Thorac Cardiovasc Surg 130: 1130–1136. 16214530 10.1016/j.jtcvs.2005.05.028

[bph14433-bib-0024] Garland CJ , Yarova PL , Jimenez‐Altayo F , Dora KA (2011). Vascular hyperpolarization to beta‐adrenoceptor agonists evokes spreading dilatation in rat isolated mesenteric arteries. Br J Pharmacol 164: 913–921. 21244369 10.1111/j.1476-5381.2011.01224.x PMC3195914

[bph14433-bib-0025] Ghatta S , Nimmagadda D , Xu X , O'Rourke ST (2006). Large‐conductance, calcium‐activated potassium channels: structural and functional implications. Pharmacol Ther 110: 103–116. 16356551 10.1016/j.pharmthera.2005.10.007

[bph14433-bib-0026] Greenstein AS , Khavandi K , Withers SB , Sonoyama K , Clancy O , Jeziorska M *et al* (2009). Local inflammation and hypoxia abolish the protective anticontractile properties of perivascular fat in obese patients. Circulation 119: 1661–1670. 19289637 10.1161/CIRCULATIONAHA.108.821181

[bph14433-bib-0027] Gurtler A , Kunz N , Gomolka M , Hornhardt S , Friedl AA , McDonald K *et al* (2013). Stain‐free technology as a normalization tool in western blot analysis. Anal Biochem 433: 105–111. 23085117 10.1016/j.ab.2012.10.010

[bph14433-bib-0028] Harding SD , Sharman JL , Faccenda E , Southan C , Pawson AJ , Ireland S *et al* (2018). The IUPHAR/BPS Guide to PHARMACOLOGY in 2018: updates and expansion to encompass the new guide to IMMUNOPHARMACOLOGY. Nucl Acids Res 46: D1091–D1106. 29149325 10.1093/nar/gkx1121 PMC5753190

[bph14433-bib-0029] Hodis J , Vaclavikova R , Farghali H (2011). β‐3 agonist‐induced lipolysis and nitric oxide production: relationship to PPARγ agonist/antagonist and AMP kinase modulation. Gen Physiol Biophys 30: 90–99. 21460417 10.4149/gpb_2011_01_90

[bph14433-bib-0031] Kilkenny C , Browne W , Cuthill IC , Emerson M , Altman DG (2010). Animal research: Reporting in vivo experiments: the ARRIVE guidelines. Br J Pharmacol 160: 1577–1579. 20649561 10.1111/j.1476-5381.2010.00872.x PMC2936830

[bph14433-bib-0033] Li R , Andersen I , Aleke J , Golubinskaya V , Gustafsson H , Nilsson H (2013). Reduced anti‐contractile effect of perivascular adipose tissue on mesenteric small arteries from spontaneously hypertensive rats: role of Kv7 channels. Eur J Pharmacol 698: 310–315. 23059186 10.1016/j.ejphar.2012.09.026

[bph14433-bib-0034] Lohn M , Dubrovska G , Lauterbach B , Luft FC , Gollasch M , Sharma AM (2002). Periadventitial fat releases a vascular relaxing factor. FASEB J 16: 1057–1063. 12087067 10.1096/fj.02-0024com

[bph14433-bib-0035] Lynch FM , Withers SB , Yao Z , Werner ME , Edwards G , Weston AH *et al* (2013). Perivascular adipose tissue‐derived adiponectin activates BK(Ca) channels to induce anticontractile responses. Am J Physiol Heart Circ Physiol 304: H786–H795. 23292715 10.1152/ajpheart.00697.2012 PMC3602769

[bph14433-bib-0036] McGrath JC , Lilley E (2015). Implementing guidelines on reporting research using animals (ARRIVE etc.): new requirements for publication in BJP. Br J Pharmacol 172: 3189–3193. 25964986 10.1111/bph.12955 PMC4500358

[bph14433-bib-0037] Minneman KP , Theroux TL , Hollinger S , Han C , Esbenshade TA (2015). Selectivity of agonists for cloned α1‐adrenergic receptor subtypes. Mol Pharmacol 46: 929–936. 7969082

[bph14433-bib-0038] Mulvany MJ , Halpern W (1977). Contractile properties of small arterial resistance vessels in spontaneously hypertensive and normotensive rats. Circ Res 41: 19–26. 862138 10.1161/01.res.41.1.19

[bph14433-bib-0039] Ng FL , Davis AJ , Jepps TA , Harhun MI , Yeung SY , Wan A *et al* (2011). Expression and function of the K+ channel KCNQ genes in human arteries. Br J Pharmacol 162: 42–53. 20840535 10.1111/j.1476-5381.2010.01027.x PMC3012405

[bph14433-bib-0040] Nguyen NL , Randall J , Banfield BW , Bartness TJ (2014). Central sympathetic innervations to visceral and subcutaneous white adipose tissue. Am J Physiol Regul Integr Comp Physiol 306: R375–R386. 24452544 10.1152/ajpregu.00552.2013 PMC3949107

[bph14433-bib-0041] Ramirez‐Ponce MP , Acosta J , Bellido JA , Mateos JC (1998). Noradrenaline modulates the electrical activity of white adipocytes by a cAMP‐dependent mechanism. J Endocrinol 159: 397–402. 9834457 10.1677/joe.0.1590397

[bph14433-bib-0042] Ramirez‐Ponce MP , Mateos JC , Carrion N , Bellido JA (1996). Voltage‐dependent potassium channels in white adipocytes. Biochem Biophys Res Commun 223: 250–256. 8670268 10.1006/bbrc.1996.0880

[bph14433-bib-0043] Rebuffe‐Scrive M (1991). Neuroregulation of adipose tissue: molecular and hormonal mechanisms. Int J Obes (Lond) 15 (Suppl. 2): 83–86. 1794942

[bph14433-bib-0044] Robidoux J , Kumar N , Daniel KW , Moukdar F , Cyr M , Medvedev AV *et al* (2006). Maximal β3‐adrenergic regulation of lipolysis involves Src and epidermal growth factor receptor‐dependent ERK1/2 activation. J Biol Chem 281: 37,794–37,802. 10.1074/jbc.M605572200 17032647

[bph14433-bib-0045] Rooks CR , Penn DM , Kelso E , Bowers RR , Bartness TJ , Harris RB (2005). Sympathetic denervation does not prevent a reduction in fat pad size of rats or mice treated with peripherally administered leptin. Am J Physiol Regul Integr Comp Physiol 289: R92–R102. 15731403 10.1152/ajpregu.00858.2004

[bph14433-bib-0046] Sansbury BE , Cummins TD , Tang Y , Hellmann J , Holden CR , Harbeson MA *et al* (2012). Overexpression of endothelial nitric oxide synthase prevents diet‐induced obesity and regulates adipocyte phenotype. Circ Res 111: 1176–1189. 22896587 10.1161/CIRCRESAHA.112.266395 PMC3707504

[bph14433-bib-0047] Saxton SN , Ryding KE , Aldous RG , Withers SB , Ohanian J , Heagerty AM (2018). Role of sympathetic nerves and adipocyte catecholamine uptake in the vasorelaxant function of perivascular adipose tissue. Arterioscler Thromb Vasc Biol 38: 880–891. 29496660 10.1161/ATVBAHA.118.310777

[bph14433-bib-0048] Schleifenbaum J , Kohn C , Voblova N , Dubrovska G , Zavarirskaya O , Gloe T *et al* (2010). Systemic peripheral artery relaxation by KCNQ channel openers and hydrogen sulfide. J Hypertens 28: 1875–1882. 20577128 10.1097/HJH.0b013e32833c20d5

[bph14433-bib-0049] Soeder KJ , Snedden SK , Cao W , Della Rocca GJ , Daniel KW , Luttrell LM *et al* (1999). The β3‐adrenergic receptor activates mitogen‐activated protein kinase in adipocytes through a Gi‐dependent mechanism. J Biol Chem 274: 12,017–12,022. 10.1074/jbc.274.17.12017 10207024

[bph14433-bib-0050] Song CK , Schwartz GJ , Bartness TJ (2009). Anterograde transneuronal viral tract tracing reveals central sensory circuits from white adipose tissue. Am J Physiol Regul Integr Comp Physiol 296: R501–R511. 19109367 10.1152/ajpregu.90786.2008 PMC2665851

[bph14433-bib-0052] Verdon CP , Burton BA , Prior RL (1995). Sample pretreatment with nitrate reductase and glucose‐6‐phosphate‐dehydrogenase quantitatively reduces nitrate while avoiding interference by NADP(+) when the Griess reaction is used to assay for nitrite. Anal Biochem 224: 502–508. 7733451 10.1006/abio.1995.1079

[bph14433-bib-0053] Verlohren S , Dubrovska G , Tsang SY , Essin K , Luft FC , Huang Y *et al* (2004). Visceral periadventitial adipose tissue regulates arterial tone of mesenteric arteries. Hypertension 44: 271–276. 15302842 10.1161/01.HYP.0000140058.28994.ec

[bph14433-bib-0054] Weston AH , Egner I , Dong Y , Porter EL , Heagerty AM , Edwards G (2013). Stimulated release of a hyperpolarizing factor (ADHF) from mesenteric artery perivascular adipose tissue: involvement of myocyte BKCa channels and adiponectin. Br J Pharmacol 169: 1500–1509. 23488724 10.1111/bph.12157 PMC3724107

[bph14433-bib-0059] Withers SB , Agabiti‐Rosei C , Livingstone DM , Little MC , Aslam R , Malik RA *et al* (2011). Macrophage activation is responsible for loss of anticontractile function in inflamed perivascular fat. Arterioscler Thromb Vasc Biol 31: 908–913. 21273560 10.1161/ATVBAHA.110.221705

[bph14433-bib-0055] Withers SB , Simpson L , Fattah S , Werner ME , Heagerty AM (2014). cGMP‐dependent protein kinase (PKG) mediates the anticontractile capacity of perivascular adipose tissue. Cardiovasc Res 101: 130–137. 24095868 10.1093/cvr/cvt229

[bph14433-bib-0056] Yamauchi T , Nio Y , Maki T , Kobayashi M , Takazawa T , Iwabu M *et al* (2007). Targeted disruption of AdipoR1 and AdipoR2 causes abrogation of adiponectin binding and metabolic actions. Nat Med 13: 332–339. 17268472 10.1038/nm1557

[bph14433-bib-0057] Zavaritskaya O , Zhuravleva N , Schleifenbaum J , Gloe T , Devermann L , Kluge R *et al* (2013). Role of KCNQ channels in skeletal muscle arteries and periadventitial vascular dysfunction. Hypertension 61: 151–159. 23184384 10.1161/HYPERTENSIONAHA.112.197566

[bph14433-bib-0058] Zhang Y , Matheny M , Zolotukhin S , Tumer N , Scarpace PJ (2002). Regulation of adiponectin and leptin gene expression in white and brown adipose tissues: influence of β3‐adrenergic agonists, retinoic acid, leptin and fasting. Biochim Biophys Acta 1584: 115–122. 12385894 10.1016/s1388-1981(02)00298-6

